# Vascular age estimation using a consumer wearable sleep tracker

**DOI:** 10.1371/journal.pdig.0001329

**Published:** 2026-03-30

**Authors:** Gizem Yilmaz, Shohreh Ghorbani, Ju Lynn Ong, Hosein Aghayan Golkashani, Chen Zhang, B. T. Thomas Yeo, Michael W. L. Chee

**Affiliations:** 1 Centre for Sleep and Cognition, Yong Loo Lin School of Medicine, National University of Singapore, Singapore, Singapore; 2 Centre for Translational Magnetic Resonance Research (TMR), Yong Loo Lin School of Medicine, National University of Singapore, Singapore, Singapore; 3 Department of Electrical and Computer Engineering, National University of Singapore, SingaporeSingapore; 4 N.1 Institute for Health and Institute for Digital Medicine (WisDM), National University of Singapore, SingaporeSingapore; 5 Integrative Sciences and Engineering Programme (ISEP), National University of Singapore, Singapore, Singapore; 6 Healthy Longevity Translational Research Programme & Human Potential Translational Research Programme, Yong Loo Lin School of Medicine, National University of Singapore, SingaporeSingapore; 7 Martinos Center for Biomedical Imaging, Massachusetts General Hospital, Boston, Massachusetts, United States of America; Henry Ford Health System, UNITED STATES OF AMERICA

## Abstract

Vascular aging is traditionally assessed using a combination of clinical markers, blood pressure and arterial stiffness measurement. However, measuring vascular aging with reference equipment is costly and not scalable. Nocturnal photoplethysmography (PPG) from wearable health trackers offer a scalable solution for longitudinal assessment. In this study, we evaluated the ability of a consumer wearable (Oura Ring) to detect age-related differences in PPG waveform, in comparison to a clinical-grade fingertip pulse oximeter. Healthy adults (N = 160; 78 males (49%), median age 31 years (IQR: 23)) underwent overnight polysomnography (PSG) in a sleep laboratory, during which fingertip and wearable ring PPG data were collected simultaneously. Pulse waveforms were extracted from both devices using a custom algorithm and key waveform features were compared across devices. Vascular age was estimated from pulse waveforms using a featureless deep learning model. Prediction performance was compared between the two devices. Age-related waveform changes were most prominent in PPG crest time (CT (samples)) (r = 0.64 and 0.62 for fingertip and wearable devices), while the reflection index (RI) had a weaker correlation with age for the ring sensor (r = 0.22) compared to fingertip (r = 0.58). Despite differences in waveforms between devices, the deep learning model showed comparable prediction performance with mean absolute errors (MAE (SD)) of 6.28 (1.48) and 7.25 (1.29) years, and r (SD) of 0.84 (0.07) and 0.80 (0.10) for clinical-grade and consumer-grade devices, respectively. These findings support the feasibility of using PPG waveforms from wearable devices to assess vascular age.

## Author summary

Vascular aging is an important marker of cardiovascular risk, traditionally assessed using clinical measurements and specialized equipment to evaluate arterial stiffness. The latter is expensive and difficult to scale for long-term or population-level monitoring. Wearable health trackers that collect nocturnal photoplethysmography (PPG) signals offer an alternative. Our study examined whether a consumer wearable device (Oura Ring) can detect age-relevant features in PPG waveforms, compared with a clinical-grade fingertip pulse oximeter. 160 healthy adults (49% male; median age 31 years) underwent overnight polysomnography, during which PPG signals were recorded simultaneously from both devices. Pulse waveforms were extracted and key waveform features were analyzed. Several PPG features showed clear age-related changes, particularly pulse crest time, which correlated strongly with age for both devices. Vascular age was then estimated using a deep-learning model applied to the PPG signals. Although waveforms differed between devices, the deep-learning model predicted vascular age with comparable accuracy using wearable and clinical-grade PPG data. Mean absolute error was approximately 6–7 years for both devices, with strong correlations between predicted and chronological age. These findings demonstrate that PPG data from consumer wearables can capture meaningful age-related vascular information, supporting their potential use for scalable, longitudinal assessment of vascular aging.

## Introduction

Modifying lifestyle risk factors is important for the prevention of cardiovascular disease, the leading cause of death in industrialized societies [1]. Vascular aging refers to age-related changes in the structure and function of the arterial system, and is an important marker of cardiovascular disease (CVD) risk [2–4]. Arterial stiffness and blood pressure, two key determinants of vascular aging, both tend to increase with age [5–8]. Unhealthy lifestyles and/or environmental agents can accelerate vascular aging [9–11]. Conversely, premature vascular aging may be mitigated through improving diet and aerobic exercise [9]. To reinforce desirable behavioural change, it would be useful to provide individuals with regular, accurate feedback through wearable health trackers, in much the same way that continuous performance monitoring has transformed sport performance.

Vascular age is commonly estimated through a vascular risk profile that incorporates established biomarkers such as blood pressure and serum lipid levels, along with arterial stiffness. Arterial stiffness is most commonly assessed as carotid–femoral pulse wave velocity (cfPWV) using applanation tonometry [2,12]. A gap between vascular and chronological age suggests early or accelerated vascular aging [3]. More recently, vascular age has been predicted using only tonometer derived arterial pulse waveforms, analyzed with a deep learning model. Vascular age predicted in this manner showed strong association with the risk of adverse cardiovascular events [13]. While this latter study demonstrated the value of analyzing arterial waveforms, a tonometry-based approach uses costly, specialized equipment and is impractical for long-term, longitudinal monitoring of arterial stiffness at scale. Approaches that enable frequent and longitudinal assessment could enhance the early identification of persons at increased risk of cardiovascular disease [4].

Wearable fitness and sleep trackers equipped with high-quality photoplethysmography (PPG) sensors are good candidates for large-scale continuous monitoring of cardiovascular health [14]. Currently, these devices primarily use PPG signals to measure pulse rate and pulse rate variability. However, high fidelity PPG waveforms can also be used to infer arterial stiffness and its age-related change [15–17]. This represents a major opportunity to longitudinally evaluate vascular health [18] and how lifestyle changes might affect arterial stiffness.

The contour of the pulse waveform, both arterial and PPG-based, changes with chronological age, as increased arterial stiffness leads to faster pulse wave propagation and enhanced wave reflection from the periphery [19,20]. With increasing age, the PPG waveform exhibits rounder systolic peaks with a lower slope, along with a less pronounced or absent dicrotic notch [15,16,21–25]. This and other PPG waveform features have been proposed to assess vascular age [26], and have been utilized in regression-based models for this purpose [27–31]. Such feature-based models rely on accurate fiducial point detection, which may be compromised by distortions in the pulse contour. An alternative is to directly feed pulse waveforms into deep learning models, which can overcome challenges in feature extraction and have demonstrated superior prediction of vascular aging compared to feature-based models [32–34]. A recent landmark study using a PPG-based deep learning framework reported associations between smartwatch-derived PPG estimates and health-related outcomes, illustrating the potential for real-world application [35]. However, PPG waveforms differ across recording site and device used and understanding which PPG features contribute to age prediction is helpful in giving insight into the underlying physiological changes that drive vascular age prediction.

In this work, we evaluated the feasibility of using a ring-based consumer device (Oura Ring) to assess vascular age using PPG waveforms and compared its performance to that of a clinical-grade, fingertip PPG sensor. Specifically, we tested whether ring derived PPG signals can comparably estimate vascular age to fingertip PPG whose signal waveforms are most familiar to clinicians. Critically, while both assess PPG at the finger, waveforms differ when obtained from the fingertip and around the finger. Assessments were conducted during sleep, which is associated with much reduced motion signal artifacts [36]. We first compared the pulse waveforms from two devices and characterized their differences. Next, we assessed the association strength between age and selected PPG waveform features. Finally, we predicted vascular age from pulse waveforms using deep learning and compared the prediction performance between the two devices.

## Results

We studied nominally healthy adults from 20 to 70 years of age who were mostly normotensive and whose characteristics are summarized in Table 1. 158 participants contributed to the PPG feature-based analysis and 160 to the deep learning prediction of vascular age.

**Table 1:** Demographic summary of samples used in PPG feature-based analysis and deep-learning based age prediction model.

Characteristic	PPG feature-based, N = 158	Deep-learning based, N = 160
**Age (years)**	31 (25)	31 (23)
20-39	108 (68.3%)	109 (68.1%)
40-59	32 (20.3%)	33 (20.6)
≥60	18 (11.4%)	18 (11.3)
**Sex (n(%))**		
**Male**	76 (48%)	78 (49%)
**Female**	82 (52%)	82 (51%)
**BMI (kg/m**^**2**^)	22.8 (3.8)	22.8 (3.9)
**SBP (mmHg)**	112 (18.9)	112 (18.7)
**DBP (mmHg)**	71 (10.5)	71 (10.4)

Median (IQR); n (%); SBP: Systolic Blood Pressure; DBP: Diastolic Blood Pressure

### Pulse waveform similarity between fingertip device and wearable ring

**Fig 1:**
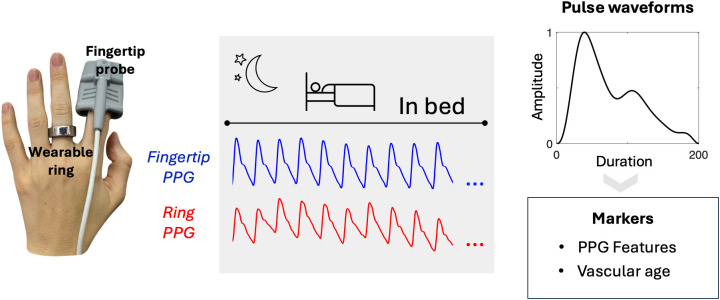
Illustration of devices, protocol and main outcomes. During sleep, participants underwent simultaneous PPG recordings using both a clinical-grade pulse oximeter (Fingertip probe) and a consumer wearable ring (Oura Ring). The data were analysed to assess PPG waveform characteristics and predict vascular age.

To determine if the wearable device retained essential pulse contour characteristics necessary for detection of relevant hemodynamic features, waveform similarity between the fingertip device and the ring pulses was evaluated (Fig 1).

**Fig 2:**
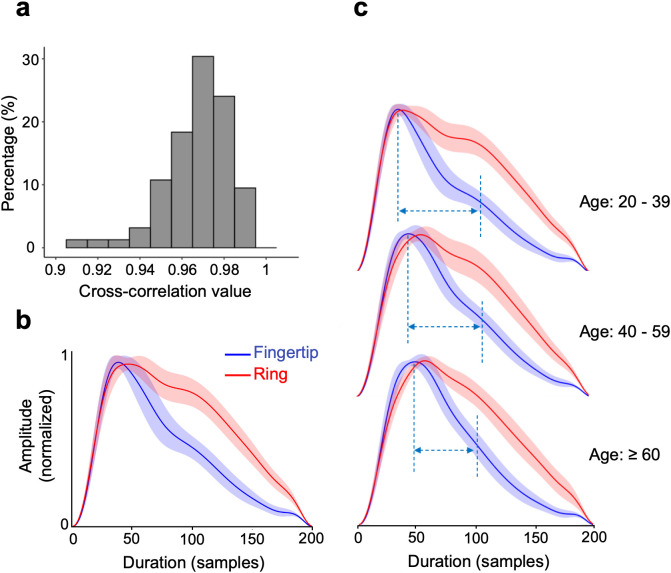
Pulse waveform similarity between the fingertip sensor and the wearable ring. **a.** Distribution of cross-correlation coefficients between devices for all participants. **b.** Average waveforms (derived from all participants) from Fingertip (blue) and Ring (red). Shaded areas show standard deviation at each sample point. **c.** Average pulse waveforms for three age groups: 20-39, 40-59, and 60 and above. Vertical dashed blue lines indicate systolic and diastolic peak locations on Fingertip pulse; horizontal dashed lines show approximate distance between 2 peaks.

The median (IQR) cross-correlation value of PPG waveforms across all participants was 0.97 (0.02) indicating overall high similarity between devices (Fig 2a). Average waveforms for the fingertip sensor and ring showed that the timing of systolic and diastolic peaks were mostly aligned, with the ring having a rounder systolic peak (Fig 2b). However, the amplitude of the diastolic phase and peak was noticeably higher in the ring compared to the fingertip sensor. With increasing age, this systolic peak showed a rounder morphology with a shift in timing, while the diastolic peak gradually flattened, giving the waveform a more blunted appearance in both devices (Fig 2c).

### Quantifying waveform differences using PPG features

Differences in waveforms between the fingertip sensor and ring were quantified using PPG features with values summarized in Table 2. Values of CT and RI differed between the devices, with the ring showing higher values. In contrast, dT did not differ significantly between the fingertip and the ring. In distribution plots, RI showed the largest difference in magnitude, with a clear separation between devices (S1 Fig). Additionally, when PPG features were not-normalized for duration, CT (seconds) was significantly higher in the ring, while values for dT (seconds) were similar (S1 Table).

**Table 2:** Summary of PPG-features from the fingertip sensor and the ring.

PPG feature	Fingertip, N = 158^1^	Ring, N = 158^1^	p-value^2^
**CT (samples)**	38.7 (9.4)	45.0 (15.3)	<0.001
**dT (samples)**	60.3 (10.6)	58.8 (10.9)	0.6
**RI (ratio)**	0.54 (0.17)	0.78 (0.12)	<0.001

^1^Median (IQR), ^2^Wilcoxon rank sum test.

CT: Crest time, dT: distance between systolic and diastolic peaks, RI: Reflection index

To examine the noise inherent in the PPG feature extraction process, we measured the agreement in PPG features between the two devices (Table 3). CT had the highest agreement between devices with correlation coefficient of 0.8, with a positive bias of 6.05 (6.16) samples indicating an overestimation for the ring. The dT and RI had similar (moderate) levels of agreement with a correlation value around 0.5, while ring overestimated RI (bias of 0.23 (0.1); S2 Fig). The degree of agreement and bias were similar for un-normalized PPG features, CT (seconds) and dT (seconds). CT (seconds) showed the best agreement between devices (S2 Table).

**Table 3:** Agreement between PPG-features extracted from the fingertip sensor and the ring.

PPG feature	Bias^1,2^	[Lower LoA, Upper LoA]	Correlation
**CT (samples)**	6.05 (6.16)	[-6.02, 18.11]	0.8
**dT (samples)**	-0.78 (7.98)	[-16.41, 14.86]	0.54
**RI (ratio)**	0.23 (0.10)	[0.04, 0.42]	0.54

^**1**^Mean (SD), ^**2**^ Bias: the difference between the ring and fingertip sensor.

LoA: Limits of Agreement, CT: Crest time, dT: distance between systolic and diastolic peaks, RI: Reflection index.

### Chronological age and pulse waveform relationship

**Fig 3:**
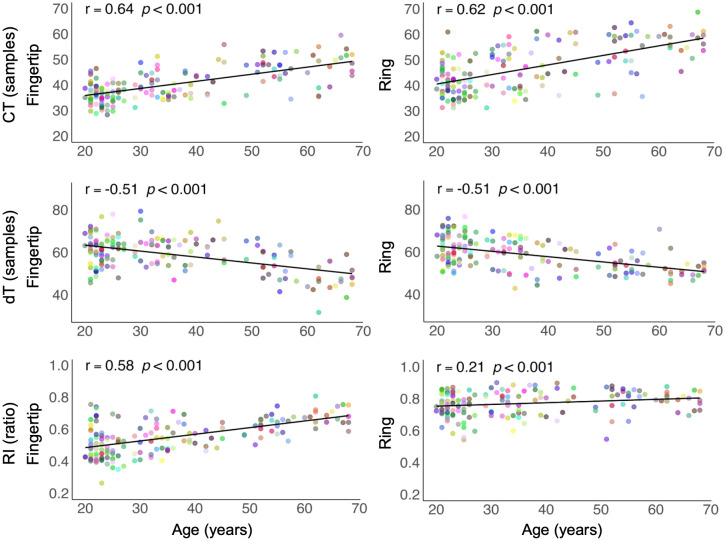
Linear relationship between age and PPG features. Scatter plots for Fingertip (left column) and Ring (right column) for CT (Crest time), dT (distance between systolic and diastolic peaks), and RI (Reflection index). Each color (point) is a different participant.

The linear relationship between chronological age and PPG features was in the expected direction for both devices (Fig 3). The strongest correlation with age was observed for CT with coefficients of 0.64 (p < 0.001) and 0.62 (p < 0.001) for fingertip and ring devices, respectively. Association with age was moderate for dT (r = -0.51, p < 0.001) for both devices. The largest discrepancy between devices was observed for RI, as correlation was 0.58 (p < 0.001) for the fingertip sensor and 0.22 (p < 0.001) for the ring. The difference in correlation strength between the two devices was significant only for RI (Fisher’s z test p < 0.05). The observed associations for PPG features and devices remained significant after correcting for sex, BMI, SBP and DBP except for RI from the ring (S3 Table). The current subset of randomly sampled windows was representative of participant means, as the correlation with age was comparable across 100 different window subsets (S3 Fig).

Lastly, in regression models predicting vascular age using CT, dT and RI alone, there was no statistical difference between prediction performance between the fingertip device and the ring (S4 Table). The regression model had a mean absolute error (MAE) of 7.76 ± 1.49 years (mean ± SD) for the fingertip device and 8.95 ± 1.54 years for the ring. The root mean square error (RMSE) was slightly lower for the fingertip device at 9.69 ± 2 years compared to 10.8 ± 1.9 years for the ring. Both devices showed moderate correlations between predicted and actual age, with correlation coefficients (r) of 0.77 ± 0.11 for the fingertip device and 0.70 ± 0.14 for the ring. Finally, R² values were 0.60 ± 0.17 for the fingertip device and 0.52 ± 0.2 for the ring.

### Age prediction via deep-learning model

Vascular age prediction performance of deep learning model (CNN) was similar between the fingertip device and the ring, with the fingertip device showing numerically slightly lower MAE and higher R² and r values (Table 4). The CNN model achieved a mean absolute error (MAE) of 6.28 ± 1.48 years (mean ± SD) for the fingertip device and 7.25 ± 1.29 years for the ring. The root mean square error (RMSE) was slightly lower for the fingertip device at 8.01 ± 1.83 years compared to 9.07 ± 1.91 years for the ring. Both devices demonstrated strong correlations between predicted and actual age, with correlation coefficients (r) of 0.84 ± 0.07 for the fingertip device and 0.80 ± 0.1 for the ring. Similarly, R² values were 0.67 ± 0.11 for the fingertip device and 0.59 ± 0.15 for the ring, indicating robust predictive performance. After adjusting for multiple comparisons, none of the differences were statistically significant between the two devices (S4 Fig and S5 Table).

**Table 4:** Age prediction performance for the fingertip sensor and the ring.

	Fingertip, N = 160^1^	Ring, N = 160^1^	p-value
**MAE**	6.28 (1.48)	7.25 (1.29)	0.080
**RMSE**	8.01 (1.83)	9.07 (1.91)	0.186
**R** ^**2**^	0.67 (0.11)	0.59 (0.15)	0.179
**r**	0.84 (0.07)	0.80 (0.10)	0.263

^1^Mean (SD), MAE: Mean absolute error, RMSE: Root mean squared error, R^2^: Coefficient of determination, r: Correlation coefficient

**Fig 4:**
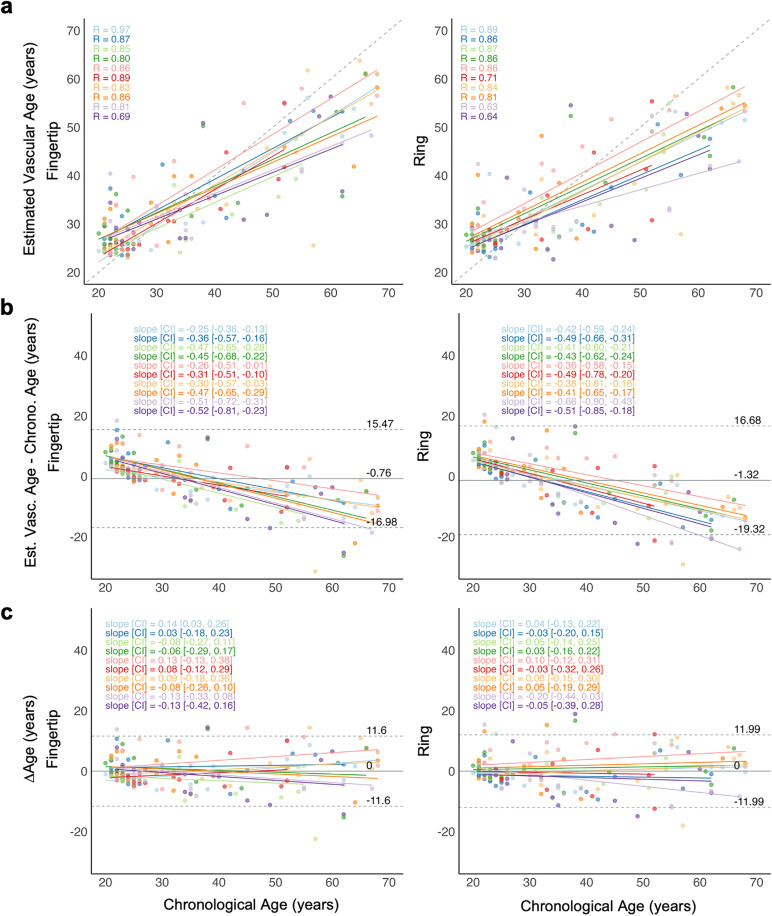
Agreement between the chronological age and estimated vascular age. **a.** Scatter plot of estimated vascular age versus chronological age across cross-validation folds for all subjects for Fingertip (left column) and Ring (right column) (N = 160). Each color represents a fold, with solid lines showing fold-specific regression fits. The dashed gray line marks perfect estimation (identity line). R: Pearson’s correlation coefficient. **b.** Bland-Altman plot comparing estimated vascular age to chronological age. The solid grey line shows mean difference, with 95% confidence intervals (dashed grey lines) representing the limits of agreement. Fold-specific regression lines and their slope and 95% CI are shown via different colors. **c.** Age-adjusted ΔAge values across chronological age after proportional bias removal. Fold-specific regression lines and their slope and 95% CI are shown via different colors.

The agreement between actual and estimated age was high for both devices, with strong correlations across the 10 folds ranging from r = 0.69 to r = 0.97 for the fingertip device, and r = 0.63 to r = 0.89 for the ring (Fig 4a). In Bland Altman plots, the mean bias (SD) was small (Fingertip: -0.75 (8.25) years, Ring: -1.31 (9.15) years) (Fig 4b). However, a pattern of proportional bias was observed with increasing age, indicating underestimation in older participants and overestimation in younger participants. The bias (raw ΔAge) was significantly associated with chronological age for both devices, with slopes [95% CI] of −0.39 [−0.45, −0.33] for the fingertip and −0.46 [−0.50, −0.40] for the ring across all folds. To visualize age-independent residual variability, age-adjusted ΔAge values are additionally presented in Fig 4c. These adjusted Bland Altman plots are intended for the characterization of variability in age-independent ΔAge values and not to assess absolute agreement.

### Key regions of the PPG waveform for CNN model

**Fig 5:**
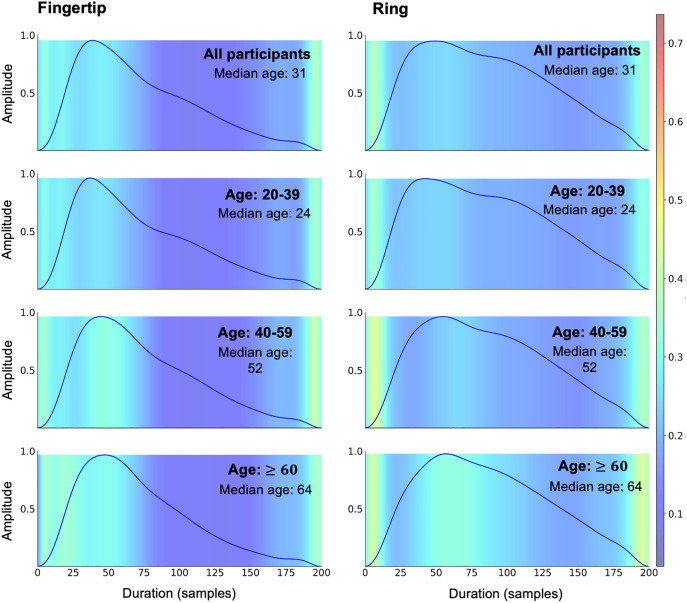
Important regions of the PPG waveform for CNN model. Grad-CAM visualizations of PPG waveforms using Fingertip (left) and Ring (right) devices. Results are displayed for different age groups across rows. Top row: all participants included, second row: ages 20–39, third row: ages 40–59, and bottom row: ages 60 and above. The colour intensity reflects the model’s attention, with warmer colours (yellow) indicating areas of higher relevance in age estimation. Across both devices, the focus areas shift with increasing age, highlighting broader regions of intermediate importance in older age groups.

Grad-CAM visualizations showed that areas of importance vary with age, with a diminished focus near the pulse onset and an expansion of intermediate-level importance regions in older age groups (ages over 60). These trends were observed for both the fingertip and the ring, suggesting that both devices capture sufficient signal features for age prediction (Fig 5).

### Associations of ΔAge with blood pressure

**Fig 6:**
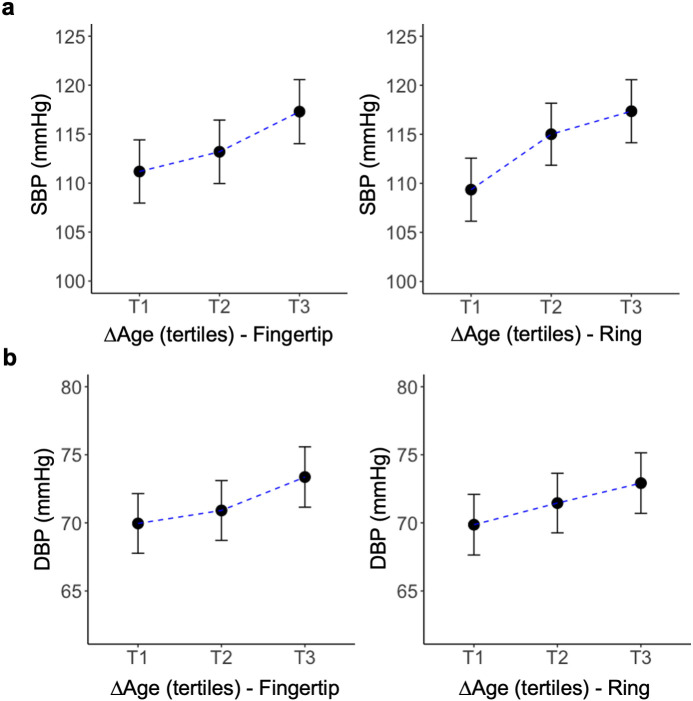
Model-adjusted systolic blood pressure (SBP) (a) and diastolic blood pressure (DBP) (b) across ΔAge tertiles derived from Fingertip and Ring. Points represent estimated marginal means from regression models adjusted for age, sex and BMI; error bars indicate 95% confidence intervals. Blue dashed lines show trend. ΔAge values are age-adjusted. Mean ΔAge for T1, T2, and T3 was −5.9, −0.5, and 6.5 years for Fingertip and −6.1, −0.4, and 6.6 years for Ring, respectively.

Model-adjusted SBP increased with increasing ΔAge grouped into tertiles (Fig 6A). For fingertip PPG, participants in the third (highest) tertile of ΔAge (T3) had significantly higher SBP than those in the first (lowest) tertile (T1) (mean difference = 6.11 mmHg, SE = 2.37, p = 0.032), while differences between T1–T2 and T2–T3 were not statistically significant. For ring PPG, compared with the first ΔAge tertile (T1), model-adjusted SBP was higher in both the second (T2; mean difference = 5.65 mmHg, p = 0.048) and third tertiles (T3; mean difference = 8.00 mmHg, p = 0.003). T2 and T3 did not differ significantly. Although DBP showed an increasing trend across ΔAge tertiles for both Fingertip and Ring, pairwise comparisons did not show statistically significant differences between groups ( Fig 6B).

## Discussion

We found that photoplethysmography (PPG) signals from a ring-based consumer device can assess vascular age as well as a clinical-grade fingertip sensor. The pulse waveforms from the ring were of comparable quality to those from the fingertip device, despite differences in pulse contours between devices. We observed moderate to high correlations with age for CT, dT, and RI when using the fingertip probe. The strength of the correlations was similar for the ring, except for RI, which had a weaker correlation with age. Finally, we predicted vascular age for both devices using raw pulse waveforms in a CNN model, achieving similar performance for both wearable and fingertip devices suggesting that the wearable ring can assess age-related changes in arterial stiffness, comparable to a fingertip sensor.

### Comparing fingertip and ring derived PPG waveforms

The observed differences in pulse waveforms between devices were primarily driven by the interdependent effects of measurement site, sensor mode and design. The ring-based wearable device used in this study measures PPG on the palmar side at the base of the finger, an area with fewer capillaries and branching arteries compared to the fingertip [37], which likely reduces sensitivity to blood volume changes and contributes to delayed systolic peak timing (i.e., a greater CT value) and a higher diastolic amplitude (i.e., a greater RI). Despite the short distance between the fingertip and the base of the finger, increased reflection at the base may have also contributed to diastolic amplification and delayed systolic timing, compared to the fingertip [38]. Additionally, the ring’s rigid form may increase contact pressure during sleep when hand swelling occurs [39], causing greater variability in temporal features (including CT and dT) with increasing contact pressure levels [40]. Lastly, the fingertip device used in this study utilized a conventional transmission-type pulse oximeter, whereas the consumer wearable uses multiple infrared LEDs in both reflectance and transmission modes to collect PPG during sleep [41]. However, a comprehensive evaluation of the effect of sensor mode on pulse contour is still needed.

With age, increased arterial stiffness leads to faster pulse wave propagation, reduced compliance of smaller peripheral arteries, and increased wave reflection [19], which in turn augments systolic pressure, steepens the upstroke, and flattens or blunts the dicrotic notch. While many features can be derived from PPG pulse waveforms, we focused on CT, dT, and RI to capture age-related changes, as these features are strongly linked to age related vascular stiffening [15,16,21–24,31,42], and are straightforward to interpret. Both the fingertip device and the ring showed a similar degree of linear association between age and CT, consistent with studies using data from the fingertip [15,23]. In contrast, the correlation for RI from the ring was significantly weaker than that from the fingertip, with only the latter consistent with previous reports [22]. The rigid structure of the ring may have caused variations in contact pressure throughout the night, potentially reducing RI’s sensitivity [40].

### PPG-based vascular age estimation models

Despite the utility of PPG features for evaluating blood vessel elasticity [26], there is currently no consensus or established guidelines on the most informative pulse characteristics for assessing vascular age. This lack of standardization becomes evident in the heterogeneity of the number and combinations of pulse waveform features used in vascular age prediction studies [30,31,33,43]. Feature-based models require precise fiducial point detection, which distortions in the pulse contour can compromise. In individuals with multiple risk factors or established cardiovascular disease, stiff arteries cause the reflected wave to merge with the direct wave, obscuring fiducial points [44]. Extracted PPG features may be even noisier with wearable data collected in real-world settings.

Using extracted pulse waveforms directly in a deep learning model may circumvent some inherent limitations of PPG feature extraction. As expected, the CNN model had better prediction performance than the PPG feature-based regression model (S5 Fig), although a more detailed comparison between the two models is beyond the scope of the current work. Nonetheless, both models showed similar performance across the two devices, and to our knowledge, these are the first findings to demonstrate that wearable PPG signals can achieve accuracy comparable to fingertip PPG in vascular age prediction. Previous deep learning studies have predicted vascular age using single-site PPG from the finger [33] and nasal cavity [30,43], multiple-site PPG from the finger, wrist, arm, and ankle [32], as well as brachial, radial, and carotid tonometry waveforms [13]. Our CNN model achieved smaller prediction errors and higher correlations than previous studies using single-site PPG [34,43], though direct comparisons are limited by differences in datasets (e.g., wake vs. sleep, sensor mode) and preprocessing pipelines. While the CNN model eliminated the need for manual feature engineering, Grad-CAM visualizations revealed that both devices relied on similar waveform regions to derive vascular age information, emphasizing consistent feature importance across devices. Notably, these highlighted regions (from onset to the dicrotic notch) align with the vascular aging characteristics of waveforms described earlier. Despite the attenuated age-related association observed for RI in the ring sensor, the CNN model captured vascular age information by leveraging the full waveform characteristics.

### Broadening the application of vascular age measurement to health monitoring

Tonometer based vascular age inference [2–4,12] is mostly confined to specialized clinics, involving infrequent measurements performed on individuals already at risk or who have cardiovascular disease. In contrast, wearable-based arterial health assessments provide a convenient means for the collection of frequent samples in the field. As these devices are widely accessible and affordable, they may prove invaluable in providing feedback about how lifestyle choices affect arterial stiffness.

Overall, our findings suggest that consumer wearables collecting high fidelity PPG data, like the Oura ring, can capture age related changes in the pulse waveform. This has significant implications for assessing vascular health unobtrusively, continuously and at scale. Consumer wearables that utilize PPG-based assessments may be instrumental in testing strategies to delay, or reverse age-associated increases in arterial stiffness by providing timely feedback about the effect of interventions. Furthermore, identifying individuals with early or accelerated vascular aging before overt CVD develops (particularly among younger or middle-aged at-risk adults who do not cross traditional treatment thresholds) could allow for pre-emptive interventions. Sleep tracking wearables in particular, offer significant advantages because sleep period is less prone motion artifacts and age-related changes in PPG waveform are more pronounced during sleep [36]. Nocturnal dipping pattern in arterial stiffness [45,46] may enable more precise characterization of vascular health compared to episodic daytime measurements, but this remains to be tested.

### Wearable vascular age estimation targets middle-aged persons interested in health preservation

While this work builds on associations between pulse waveform morphology, aging, and arterial stiffness to explore wearable-based vascular age assessment, there are two important points to note. The first concerns the disproportionate contribution of younger and mid-age persons to the data that is intended to inform about ‘vascular aging’. The distribution of users reported here is typical of the user base of wearable trackers, i.e., mid-aged and younger persons. These persons often purchase devices for health improvement or preservation and stand to gain the most from desirable lifestyle modifications in contrast to older adults who may already have accumulated pathology. Second, the CNN model’s performance and the proportional bias could potentially be improved by training on a larger, more demographically and clinically diverse population. However, proportional bias, where prediction error increases systematically with the magnitude of the measured variable, is a well-documented phenomenon in organ age prediction studies using regression-based deep learning models [47,48]. The age dependence of the raw age gap (raw-ΔAge) can be attenuated by regressing raw-ΔAge on chronological age to adjust for regression-to-the-mean bias [35,47–49]. This adjustment is particularly relevant when assessing associations between organ age estimates (e.g., vascular age) and clinical outcomes. Adjusted ΔAge values are shown in Fig. 4c to illustrate the reduction of proportional bias and to visualize variability in age-independent ΔAge values.

Relatedly, it is crucial that deviations from chronological age should not be viewed solely as a prediction error, as our findings suggest they may reflect inter-individual differences in vascular age that relate to true differences in cardiovascular risk. Even in our predominantly healthy sample, BP tended to increase with increasing ΔAge (Fig. 6) and the relationship was more pronounced for SBP. We refrain from over-interpreting these results as our sample had restricted range of BP values. These effect sizes could be larger in populations at higher cardiovascular risk. Testing PPG-based vascular age prediction against the cfPWV-based approach and in less healthy participants is an important direction for future work.

Data from multiple nights would be helpful to assess the reliability and stability of the current prediction results, as well as to evaluate possible changes in arterial stiffness in accordance to lifestyle modifications. In cases where there were 2 nights from the same participant, the correlation between estimated vascular ages between night 1 and night 2 was strong for both the Fingertip and Ring devices, although the Ring showed slightly lower correlation (S6 Fig). Currently, vascular age is clinically assessed during a single clinic visit. Understanding how PPG waveform characteristics, and by extension, vascular age, fluctuates from measurement to measurement over a short time interval remains an important question.

## Conclusion

In summary, in an aging population, scalable methods to identify individuals with early or accelerated vascular aging is essential. We demonstrate that PPG signals from consumer wearable can reasonably estimate vascular age, facilitating scalable monitoring of vascular health in the field. Given the strong link between arterial stiffness and disease, the findings of this study hold great promise for preventive healthcare.

## Methods

### Ethics declaration

All procedures were approved by the Institutional Review Board of the National University of Singapore (NUS-IRB-2020–463), and the protocol was in accordance with the principles in the Declaration of Helsinki. All participants signed written informed consent before commencing the study.

### Study design and participants

Nominally healthy adults aged between 20 and 70 were recruited for two separate overnight sleep studies conducted one year apart [36,50]. A total of 165 healthy adults provided PPG data, of whom 160 were included in the analysis [(78 (49%) males; median (IQR) age 31(23), Table 1)]. Participants were free from pre-existing sleep, neurological or psychiatric disorders, excessive daytime sleepiness (Epworth Sleepiness Scale 43 scores > 10), not on wake-promoting medications, had a body mass index (BMI) < 35 kg/m2 and habitually slept more than 5 hours/night.

Demographic information, height, weight and office blood pressure (BP), were collected during a daytime briefing session prior to their overnight sleep sessions. Office BP was measured three times per participant, and the average of the three readings was used as the final BP value. In addition, participants completed questionnaires regarding their history of medically diagnosed hypertension and current medication use. Informed consent was obtained during the briefing session.

Based on self-reports, 2 participants had hypertension, with 1 on antihypertensive medication. Only 4 out of 160 participants met the Singapore hypertension criteria (SBP ≥ 140 mmHg or DBP ≥ 90 mmHg) based on office BP measurements (S7 Fig).

Participants slept overnight in the lab while polysomnography (PSG), fingertip photoplethysmography (PPG) and wearable ring data were collected simultaneously. Lights off and lights on times were assigned according to the participant’s habitual bed and wake times.

## Data acquisition

### Polysomnography (PSG)

As part of the polysomnography (PSG) setup (SOMNOmedics GmbH, Randersacker, Germany), electroencephalography was recorded from two channels (C3 and C4) according to the international 10–20 system, and referenced to the contralateral mastoids. The common ground and reference electrodes were placed at Fpz and Cz, respectively. Electrooculography (EOG; right and left outer canthi), submental electromyography (EMG), thoracic breathing effort, electrocardiography (ECG) and plethysmography (PPG) signals were recorded. The sampling rate of EEG, ECG, and PPG was 256 Hz. PSG recordings for the entire night were segmented into 30-second epochs between “lights off” and “lights on” markers (denoting when participants were in bed) for PPG pulse extraction (see below).

### Fingertip pulse oximeter

The fingertip PPG signal was recorded using the pulse oximeter probe of a SOMNOtouch device (SOMNOmedics GmbH, Randersacker, Germany) which operates on a transmission mode using red (660 nm) and infrared (880nm) light emitting diodes (LEDs) [51]. Sampling rate was 256 Hz. The PPG sensor was placed on the finger that provided the best fit (often the index finger) of the non-dominant hand.

### Consumer Wearable Ring

The Oura Ring Gen 3 (Oura Ring Inc, Oulu, Finland) is a commercially available sleep, pulse rate and activity tracker. It employs multiple infrared (900 nm) LEDs on partially transmissive and partially reflective modes concurrently to collect PPG during sleep [41]. For this study, Oura Ring Inc provided “memory rings” with the same sensors as the commercially available Oura Ring, but with additional flash memory to record minimally processed sensor data. The ring was worn on the non-dominant hand but on a different finger (than the fingertip probe) that provided the best fit. Sampling rate of the PPG was 50 Hz. Fig 1 illustrates placement of devices and the PPG collection protocol.

### Pulse waveform analysis

Pulse waveform and feature extraction were applied to the night-level data of each participant. After bandpass filtering (0.05 - 20 Hz, 4th order Chebyshev) and detection of large motion artefacts (via signal envelope based thresholding), the PPG signal was segmented into 30-second windows between light off and on times. Within these 30 sec windows, signal segments were analysed to find individual pulse waveforms and extract feature information.

Initially, pulses (onset times) were detected using the qppg.m function from The PhysioNet Cardiovascular Signal Toolbox [52]. Artefactual onset detections were automatically removed based on physiological plausibility criteria: 1) Lower and upper limits of onset-to-onset duration were set to 0.4 sec and 2 sec, 2) Maximum acceptable change in peak-to-peak duration between consecutive pulses was set to 40%. Baseline drift on the PPG segment was removed using cubic spline [53,54]. Individual pulses were normalized in width into 200 equally spaced intervals by cubic spline interpolation [55]. Pulse amplitude was normalized to a range of 0–1. Only pulses that passed the signal quality check were used in the feature extraction step and age prediction model (See S8 Fig).

Fiducial points on the pulse wave (onset, offset, systolic peak, diastolic peak, and the dicrotic notch) were identified using a custom algorithm that used the first, second and third derivatives of the pulse together with peak information from the fitted gaussians on the template (See S9 Fig). Using fiducial points, we derived the following indices, which were found to be associated with age: *CT (crest time)* as the time from onset to systolic peak [15]; *dT* as the time difference between systolic and diastolic peaks [16]; *RI (reflection index)* as the ratio between diastolic and systolic peak amplitudes [22]. To account for the influence of heart rate (HR) variations, individual pulses were normalized in width to 200 equally spaced intervals using cubic spline interpolation [36], and unless otherwise stated, results for duration-normalized pulses and PPG features (CT, dT and RI) are presented in the main text, while results for un-normalized pulses (CT and dT with duration in seconds) are provided in the supplementary for comparison with previous studies. Since RI is a ratio of amplitudes, normalization did not affect values obtained. The median value of PPG features was calculated for each 30-second window, and used in paired analysis.

### Data inclusion

Pre-processed night-level data was grouped by participant and concatenated into a single participant-level data frame, as some participants had data from two sleep sessions [36]. For waveform similarity and PPG feature analysis (feature-based model), Fingertip and Oura data were paired at the 30-second window level, with twenty pairs randomly selected per participant. Participant-level data for each sensor was averaged across paired windows, resulting in 158 participants being included. The median (IQR) number of pulses per participant (used in waveform comparison and PPG-features analysis) was 364 (152) for Fingertip and 367 (139) for Ring (S10 Fig-b).

For the deep-learning model, number of pulses was standardized by randomly selecting 370 pulses per participant from each device. This value was chosen based on inspection of the per-participant pulse count distributions for both sensors and to ensure consistency with the PPG feature–based analyses. This resulted in a dataset with 160 participants for the deep learning model (See S10 Fig-a)

### Vascular age estimation (regression model)

To assess the accuracy of vascular age prediction using PPG features alone, we built multiple linear regression models with CT, dT, and RI as predictors and age as dependent variable. A 10-fold cross-validation approach was used, with a 90/10 train-test split. Model performance was evaluated on the test set of each fold, and the results were reported as the mean performance across all 10 folds.

### Vascular age estimation (deep-learning model)

The deep learning framework consisted of a 1D convolutional neural network (CNN) followed by fully connected (FC) layers. We used a compact architecture (two convolutional layers followed by two FC layers) to limit model capacity and reduce overfitting risk given the participant-level sample size (N = 160). This design was informed by prior work [34] which used fixed-length PPG pulse inputs, moderate kernel sizes with stride 1, and a 1024-unit FC layer with dropout, and explored different convolutional depths. In our exploratory experiments, deeper variants (3–4 convolutional layers) did not improve cross-validated validation performance; therefore, we retained the two-layer model for robustness and reproducibility. The network input was a single-channel PPG pulse resampled from onset to offset into a fixed-length 200 × 1 vector. The two convolutional layers used 16 filters (kernel size 11) and 32 filters (kernel size 9), with stride = 1 and no pooling, followed by two FC layers (1024 units) and dropout (p = 0.2) applied after each FC layer, and a final linear output neuron. Stacking the 11- and 9-sample kernels yields an effective receptive field of 19 samples (~9.5% of the pulse), providing local context for morphology learning. ReLU activations were applied throughout (S8 Fig).

### Training and optimization

A ten-fold cross-validation approach was used for model optimization and evaluation. The folds were constructed at the participant level. In each iteration, one fold (16 participants) served as the test set. The remaining nine folds were split into training and validation sets in an 8:1 ratio, yielding 128 participants in the training set and 16 participants in the validation set. Care was taken so that waveforms of the same participant were exclusively assigned to either training, validation or test set within each fold.

Hyperparameter optimization was conducted separately for each fold’s validation set. A heuristic grid search was performed for each fold, exploring various hyperparameter configurations listed in S6 Table. For the SGD optimizer, the gradient decay rates (β₁ and β₂) were 0.9 and 0.999 and the weight decay value for both Adam and SGD optimizers was 1 × 10^-7^. The final model performance was evaluated on the test set of each fold using the optimal hyperparameter set of that fold.

### grad-cam visualisation

Grad-CAM (Gradient-weighted Class Activation Mapping) is a technique for visualizing the regions of an input that significantly influence a CNN’s predictions [56]. Heatmaps were generated to identify the regions of the PPG waveform most relevant to the model’s predictions. These heatmaps were averaged across all pulses and stratified by different age groups to examine age-related patterns in the PPG signals.

### Statistical analysis

Variables were summarized as mean ± SD or median (IQR) based on their distribution, and appropriate parametric or non-parametric tests were applied. Fisher’s z-test was used to compare linear correlation coefficients. As part of sensitivity analyses, random sampling of paired windows was repeated for 100 times, and relationship between age and PPG-features was evaluated for each cycle. This was done to show that randomly sampled windows were representative of participant-mean and not dominated by any sleep stage (particularly as deep sleep tends to dominate in the earlier part of the night). To assess whether the performance metrics (MAE, RMSE, R², and correlation coefficient) differed significantly between Fingertip and Ring across the 10-fold cross-validation results, we employed the corrected resampled t-test [57,58]. Bonferroni correction was applied for multiple comparisons. In Bland Altman analysis, raw bias or raw-ΔAge was defined as the difference between the estimated vascular age and chronological age. The limits of agreement (LoA) were defined as mean bias ± 1.96 × SD of the differences. A linear regression model was fitted with raw-ΔAge as the dependent variable and chronological age as the independent variable. The residuals from this regression were used as age-adjusted ΔAge values [47,48]. Lastly, age-adjusted ΔAge values were grouped into tertiles for both Fingertip and Ring, and their associations with BP were examined using linear regression models including age, sex, and BMI as covariates. Bonferroni correction was applied to account for multiple comparisons.

### Code Availability

PPG pulse waveform and feature extraction were conducted in MATLAB version R2023b (The Math Works, Inc., Natick, MA) using custom algorithms. The signal quality criteria and data exclusion rules were identical for both the fingertip and the ring PPG. The deep learning model was implemented and executed using Python 3.10.15, PyTorch 2.0.1 with CUDA 11.7 (compiler version of V11.7.64.) Model training and validation were conducted on a high-performance computing (HPC) system equipped with four NVIDIA GeForce RTX 3090 GPUs. The GPU setup was managed by NVIDIA-SMI (Driver Version: 550.54.14). Code used in this study can be accessed at https://github.com/SCL-NUS/ppg-vascularage-ring.

## Supporting information

S1 Fig Distributions of PPG features (duration normalized) for Fingertip and Ring, across participants. RI showed the largest difference in magnitude, with a clear separation between devices, while CT and dT were overlapping. Red color is used for Ring, blue is for Fingertip. CT: Crest time, dT: distance between systolic and diastolic peaks, RI: Reflection index.
(DOCX)

S2 Fig Agreement between PPG features from the fingertip sensor and the ring. The scatter plots for Fingertip and Ring PPG features are on the left column, while Bland-Altman plots are on the right. Each color represents a unique participant. (r: pearson’s correlation coefficient, ccc: Lin’s correlation coefficient), CT: Crest time, dT: distance between systolic and diastolic peaks, RI: Reflection index.
(DOCX)

S3 Fig Distribution of correlation coefficients between age and PPG features for 100 different subsets of 20 randomly sampled windows used to calculate participant means. Vertical dashed lines indicate group means, and the text above the lines shows mean (SD). Blue represents Fingertip, and red represents Ring. CT: Crest time; dT: distance between systolic and diastolic peaks; RI: Reflection index.
(DOCX)

S4 Fig Prediction accuracy comparison between devices. Results with corrected resampled paired t-test for dependencies between 10 folds. P-values are corrected for multiple comparisons.
(DOCX)

S5 Fig Comparison of model performance between CNN and regression models for Fingertip (A) and Ring (B). Each dot represents a fold. REG: Regression.
(DOCX)

S6 Fig Correlation of estimated vascular age between Night 1 and Night 2 for Fingertip (A) and Ring (B). Each dot represents a subject. Only 63 participants had 2 nights of data (N = 63). The trained models and the original participant assignments to the training, validation, and test sets were kept unchanged. New test sets were created by selecting participants with two nights of data from each fold’s test set and randomly sampling 150 pulses from each night. The trained models were then applied separately to the Night 1 and Night 2 data to predict vascular age. r: Pearson correlation coefficient.
(DOCX)

S7 Fig Distribution of systolic (SBP) and diastolic (DBP) blood pressure values among participants. Each dot represents a participant, with color indicating chronological age. Red dashed lines indicate blood pressure values for hypertension as defined in Singapore (SBP ≥ 140 mmHg, DBP ≥ 90 mmHg), the red rectangle in the top-right corner highlights the hypertension range.
(DOCX)

S8 Fig Summary of analysis pipeline. Preprocessing and age prediction using deep learning model are illustrated. QC: Quality Check, FC: Fully Connected.
(DOCX)

S9 Fig Summary of fiducial point detection algorithm using pulse and its derivatives. Fiducial points are detected as follows: Onset: 1st sample on the waveform. Offset: Last sample on the waveform. Systolic peak: Point where 1st derivative crosses zero in positive to negative direction for the 1st time. Estimated diastolic peak: The peak of the second gaussian (which was fitted during template creation) was used as an estimated diastolic peak by adding %10 of it’s distance from the onset. Dicrotic notch: In the 3rd derivative signal, the nearest zero-crossing in the positive-to-negative direction closest to the estimated diastolic peak. Real diastolic peak: In the 1st derivative signal, the positive-to-negative zero-crossing within the segment between the dicrotic notch and the estimated diastolic peak.
(DOCX)

S10 Fig Distribution of total number of pulses. a. After preprocessing, 165 participants had at least one 30-second window of high-quality data from both Fingertip (blue) and Ring (red), before applying data inclusion criteria. Dashed black line marks the number of pulses included into age prediction model (370 pulses). For the included 160 participants (with at least 370 pulses from each device), the median (IQR) number of total pulses per participant was 11,988 (11,092) from Fingertip and 11,727 (10,000) from Ring. b. Distribution of number of pulses included into waveform comparison across 158 participants for Fingertip (blue) and Ring (red). The median (IQR) number of pulses per participant (used in waveform comparison and PPG-features analysis) was 364 (152) for Fingertip and 367 (139) for Ring.
(DOCX)

S1 Table Summary of PPG-features (un-normalized duration, in seconds). When PPG features were not-normalized for duration, CT (seconds) was significantly higher in the ring, while values for dT (seconds) were similar.
(DOCX)

S2 Table Agreement between un-normalized PPG-features (in seconds). Bias is calculated as the difference between the ring and the fingertip sensor. LoA refers to Limits of Agreement. CT: Crest time, dT: distance between systolic and diastolic peaks, PW: Pulse width.
(DOCX)

S3 Table Regression models to predict CT, dT and RI for the fingertip sensor and the ring. (DOCX)

S4 Table Age prediction performance of the PPG-based regression model using the fingertip sensor and the ring. (DOCX)

S5 Table Prediction performance for all test sets for Fingertip and Ring. (DOCX)

S6 Table Hyperparameter values used in optimization and evaluation. (DOCX)
