# Supplementary material for: Vascular age estimation using a consumer wearable sleep tracker
Source: PLOS Digit Health. 2026 Mar 30;5(3):e0001329. doi: 10.1371/journal.pdig.0001329 (PMC13035161; doi:10.1371/journal.pdig.0001329)
Supplement: S1 Fig — RI showed the largest difference in magnitude, with a clear separation between devices, while CT and dT were overlapping. Red color is used for Ring, blue is for Fingertip. CT: Crest time, dT: distance between systolic and diastolic peaks, RI: Reflection index. (DOCX) [file pdig.0001329.s001.docx]

**S1 Fig.** **Distributions of PPG features (duration normalized) for Fingertip and Ring, across participants.** RI showed the largest difference in magnitude, with a clear separation between devices, while CT and dT were overlapping. Red color is used for Ring, blue is for Fingertip. CT: Crest time, dT: distance between systolic and diastolic peaks, RI: Reflection index
