# Supplementary material for: Vascular age estimation using a consumer wearable sleep tracker
Source: PLOS Digit Health. 2026 Mar 30;5(3):e0001329. doi: 10.1371/journal.pdig.0001329 (PMC13035161; doi:10.1371/journal.pdig.0001329)
Supplement: S2 Fig — The scatter plots for Fingertip and Ring PPG features are on the left column, while Bland-Altman plots are on the right. Each color represents a unique participant. (r: pearson’s correlation coefficient, ccc: Lin’s correlation coefficient), CT: Crest time, dT: distance between systolic and diastolic peaks, RI: Reflection index. (DOCX) [file pdig.0001329.s002.docx]

**S2 Fig.** **Agreement between PPG features from the fingertip sensor and the ring.** The scatter plots for Fingertip and Ring PPG features are on the left column, while Bland-Altman plots are on the right. Each color represents a unique participant. (r: pearson’s correlation coefficient, ccc: Lin’s correlation coefficient), CT: Crest time, dT: distance between systolic and diastolic peaks, RI: Reflection index
