# Supplementary material for: Vascular age estimation using a consumer wearable sleep tracker
Source: PLOS Digit Health. 2026 Mar 30;5(3):e0001329. doi: 10.1371/journal.pdig.0001329 (PMC13035161; doi:10.1371/journal.pdig.0001329)
Supplement: S3 Fig — Vertical dashed lines indicate group means, and the text above the lines shows mean (SD). Blue represents Fingertip, and red represents Ring. CT: Crest time; dT: distance between systolic and diastolic peaks; RI: Reflection index. (DOCX) [file pdig.0001329.s003.docx]

**S3 Fig**: **Distribution of correlation coefficients between age and PPG features for 100 different subsets of 20 randomly sampled windows used to calculate participant means.** Vertical dashed lines indicate group means, and the text above the lines shows mean (SD). Blue represents Fingertip, and red represents Ring. CT: Crest time; dT: distance between systolic and diastolic peaks; RI: Reflection index.
