# Supplementary material for: Vascular age estimation using a consumer wearable sleep tracker
Source: PLOS Digit Health. 2026 Mar 30;5(3):e0001329. doi: 10.1371/journal.pdig.0001329 (PMC13035161; doi:10.1371/journal.pdig.0001329)
Supplement: S4 Fig — Results with corrected resampled paired t-test for dependencies between 10 folds. P-values are corrected for multiple comparisons. (DOCX) [file pdig.0001329.s004.docx]

**S4 Fig. Prediction accuracy comparison between devices.** Results with corrected resampled paired t-test for dependencies between 10 folds. P-values are corrected for multiple comparisons.
