# Supplementary material for: Vascular age estimation using a consumer wearable sleep tracker
Source: PLOS Digit Health. 2026 Mar 30;5(3):e0001329. doi: 10.1371/journal.pdig.0001329 (PMC13035161; doi:10.1371/journal.pdig.0001329)
Supplement: S5 Fig — Each dot represents a fold. REG: Regression. (DOCX) [file pdig.0001329.s005.docx]

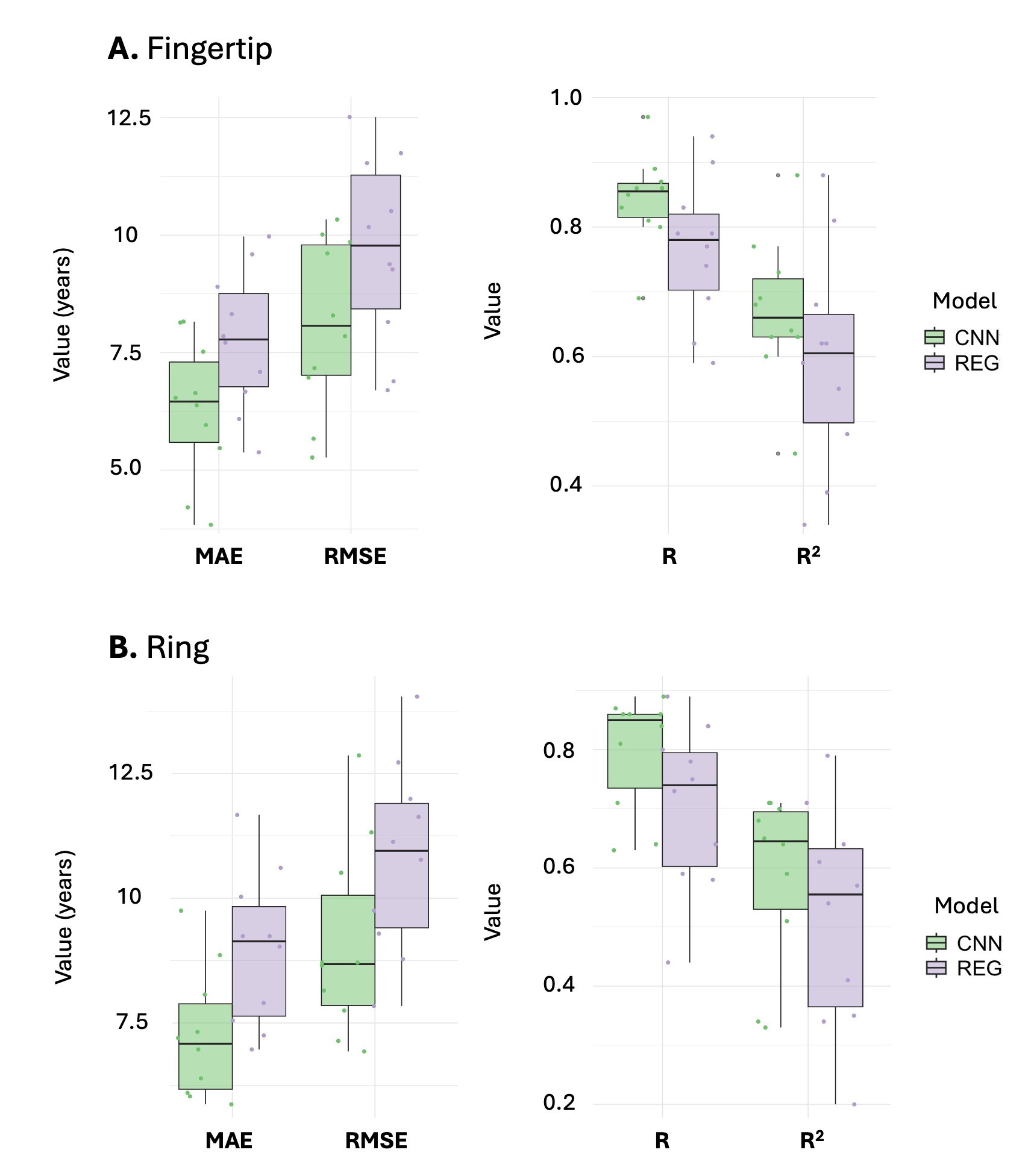


**S5 Fig. Comparison of model performance between CNN and regression models for Fingertip (A) and Ring (B).** Each dot represents a fold. REG: Regression

**Study design and participants**


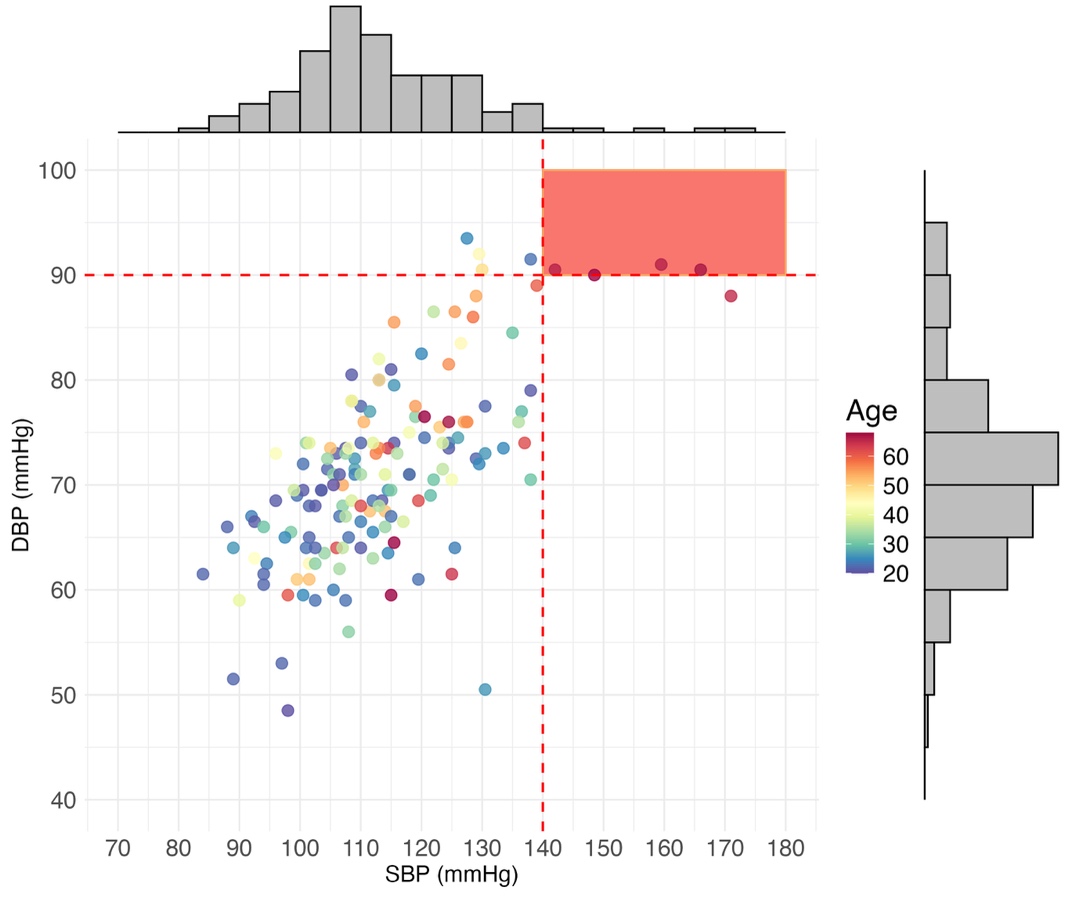


**S6 Fig.** **Distribution of systolic (SBP) and diastolic (DBP) blood pressure values among participants.** Each dot represents a participant, with color indicating chronological age. Red dashed lines indicate blood pressure values for hypertension as defined in Singapore (SBP ≥ 140 mmHg, DBP ≥ 90 mmHg), the red rectangle in the top-right corner highlights the hypertension range.

**PPG waveform analysis**

**S7 Fig. Summary of analysis pipeline.** Preprocessing and age prediction using deep learning model are illustrated. QC: Quality Check, FC: Fully Connected.

**Fiducial points detection on PPG pulse waveform**

**S8 Fig.** **Summary of fiducial point detection algorithm using pulse and its derivatives**. Fiducial points are detected as follows: Onset: 1^st^ sample on the waveform. Offset: Last sample on the waveform. Systolic peak: Point where 1^st^ derivative crosses zero in positive to negative direction for the 1^st^ time. Estimated diastolic peak: The peak of the second gaussian (which was fitted during template creation) was used as an estimated diastolic peak by adding %10 of it’s distance from the onset. Dicrotic notch: In the 3^rd^ derivative signal, the nearest zero-crossing in the positive-to-negative direction closest to the estimated diastolic peak. Real diastolic peak: In the 1st derivative signal, the positive-to-negative zero-crossing within the segment between the dicrotic notch and the estimated diastolic peak.

**Data inclusion criteria and distribution of pulses**

**S9 Fig.** **Distribution of total number of pulses.** **a.** After preprocessing, 165 participants had at least one 30-second window of high-quality data from both Fingertip (blue) and Ring (red), before applying data inclusion criteria. Dashed black line marks the number of pulses included into age prediction model (370 pulses). For the included 160 participants (with at least 370 pulses from each device), the median (IQR) number of total pulses per participant was 11,988 (11,092) from Fingertip and 11,727 (10,000) from Ring. **b.** Distribution of number of pulses included into waveform comparison across 158 participants for Fingertip (blue) and Ring (red). The median (IQR) number of pulses per participant (used in waveform comparison and PPG-features analysis) was 364 (152) for Fingertip and 367 (139) for Ring.

**Deep learning model hyperparameters**

**S6 Table.** **Hyperparameter values used in optimization and evaluation**.

| **Hyperparameter** | **Compared options (Test)** |
| --- | --- |
| **Dropout Rate** | 0.2, 0.4 |
| **Learning Rate** | 0.01, 0.001, 0.0001, 0.00001 |
| **Activation Function** | ReLU |
| **Optimizer** | SGD, Adam |
| **Loss Function** | L1, MSE |
| **Batch size** | 128, 256 |
| **Epoch size** | 200, 300, 500 |

**Night to night variability**


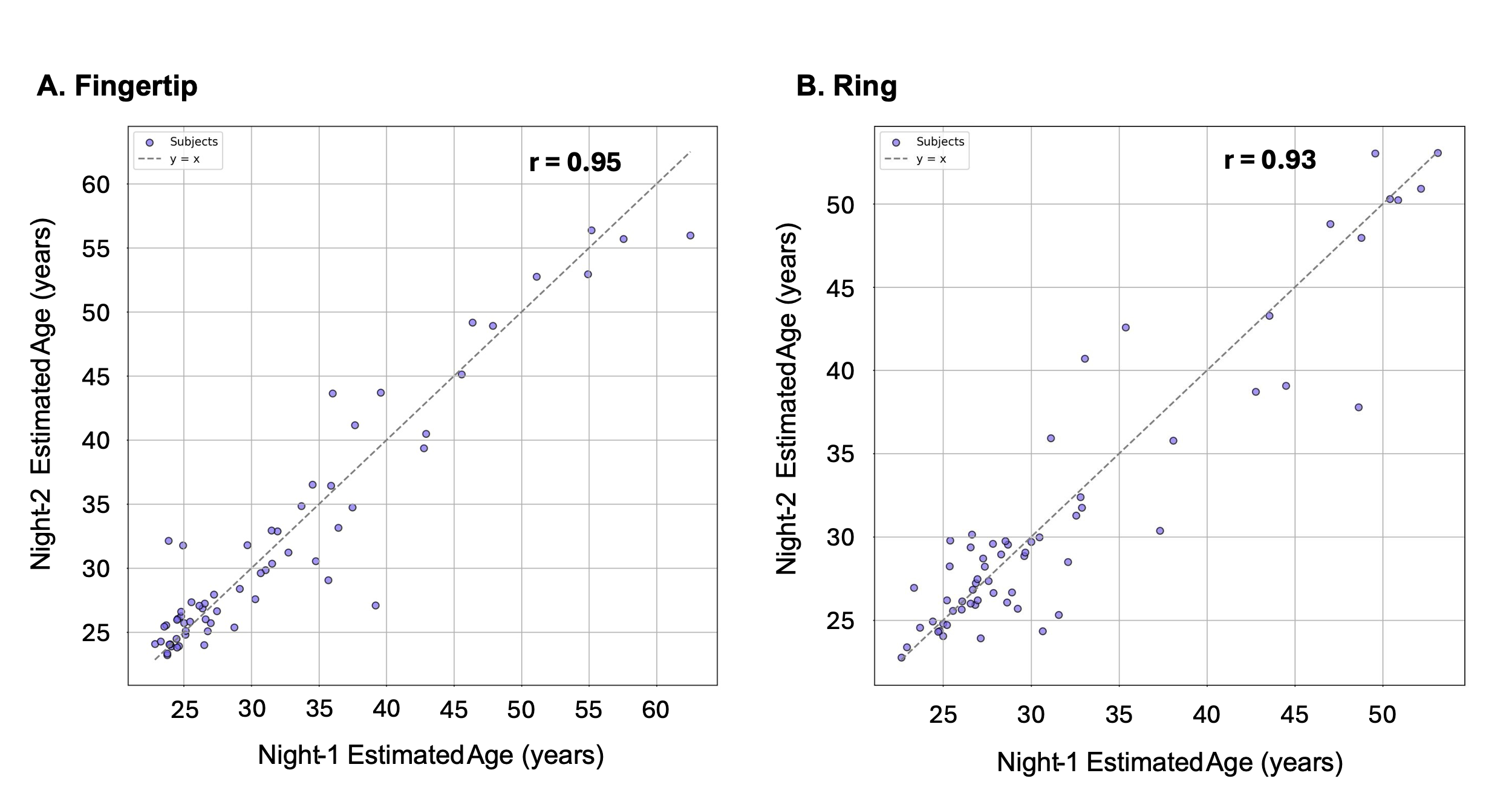


**S10 Fig.** **Correlation of estimated vascular age between Night 1 and Night 2 for Fingertip (A) and Ring (B).** Each dot represents a subject. Only 63 participants had 2 nights of data (N=63). The trained models and the original participant assignments to the training, validation, and test sets were kept unchanged. New test sets were created by selecting participants with two nights of data from each fold’s test set and randomly sampling 150 pulses from each night. The trained models were then applied separately to the Night 1 and Night 2 data to predict vascular age. r: Pearson correlation coefficient.
