# Supplementary material for: Vascular age estimation using a consumer wearable sleep tracker
Source: PLOS Digit Health. 2026 Mar 30;5(3):e0001329. doi: 10.1371/journal.pdig.0001329 (PMC13035161; doi:10.1371/journal.pdig.0001329)
Supplement: S6 Fig — Each dot represents a subject. Only 63 participants had 2 nights of data (N = 63). The trained models and the original participant assignments to the training, validation, and test sets were kept unchanged. New test sets were created by selecting participants with two nights of data from each fold’s test set and randomly sampling 150 pulses from each night. The trained models were then applied separately to the Night 1 and Night 2 data to predict vascular age. r: Pearson correlation coefficient. (DOCX) [file pdig.0001329.s006.docx]

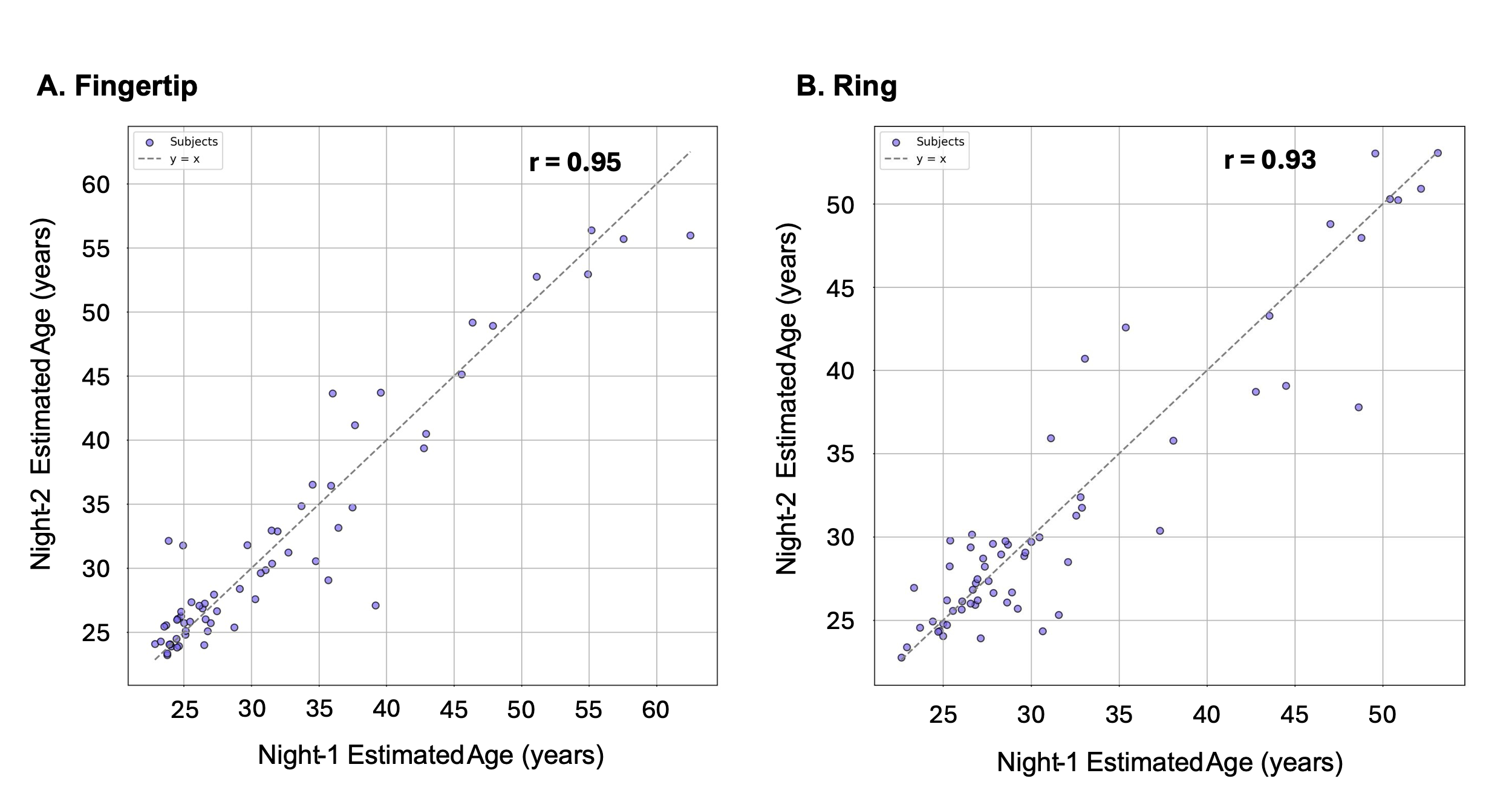


**S6 Fig.** **Correlation of estimated vascular age between Night 1 and Night 2 for Fingertip (A) and Ring (B).** Each dot represents a subject. Only 63 participants had 2 nights of data (N=63). The trained models and the original participant assignments to the training, validation, and test sets were kept unchanged. New test sets were created by selecting participants with two nights of data from each fold’s test set and randomly sampling 150 pulses from each night. The trained models were then applied separately to the Night 1 and Night 2 data to predict vascular age. r: Pearson correlation coefficient.
