# Supplementary material for: Vascular age estimation using a consumer wearable sleep tracker
Source: PLOS Digit Health. 2026 Mar 30;5(3):e0001329. doi: 10.1371/journal.pdig.0001329 (PMC13035161; doi:10.1371/journal.pdig.0001329)
Supplement: S7 Fig — Each dot represents a participant, with color indicating chronological age. Red dashed lines indicate blood pressure values for hypertension as defined in Singapore (SBP ≥ 140 mmHg, DBP ≥ 90 mmHg), the red rectangle in the top-right corner highlights the hypertension range. (DOCX) [file pdig.0001329.s007.docx]

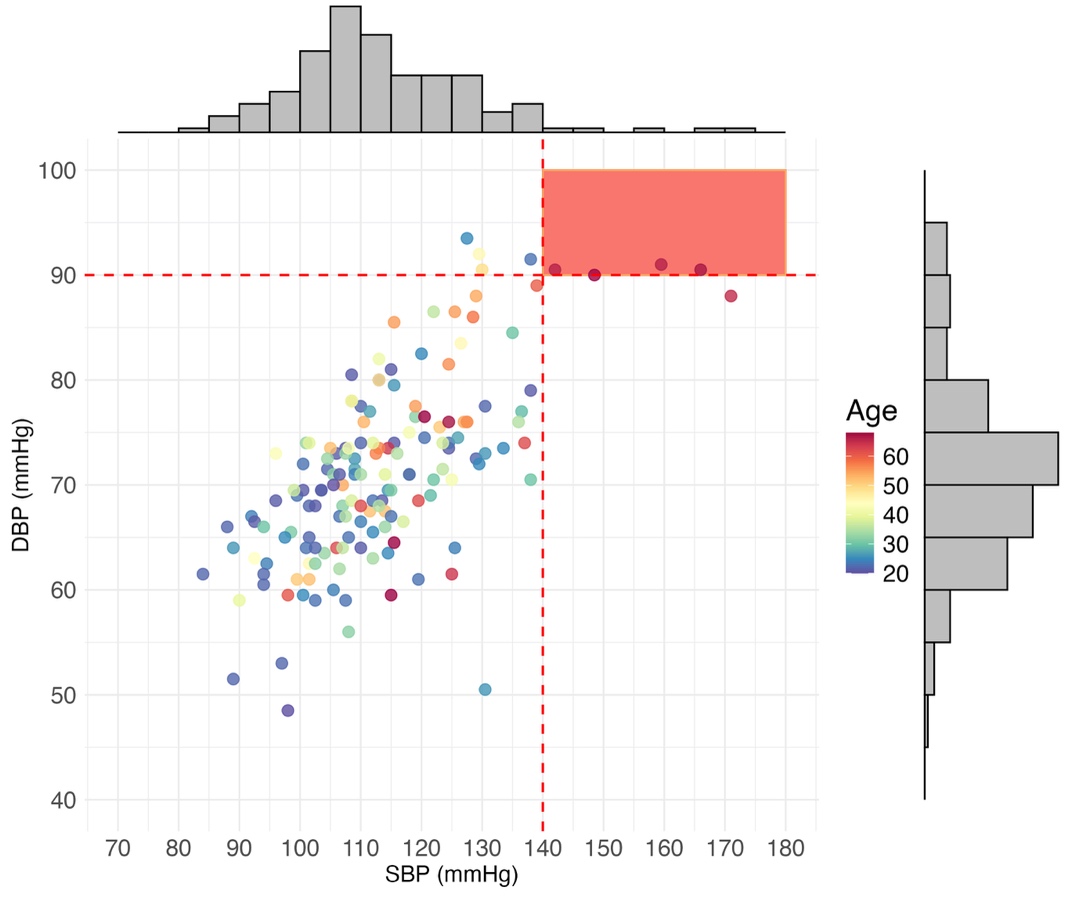


**S7 Fig.** **Distribution of systolic (SBP) and diastolic (DBP) blood pressure values among participants.** Each dot represents a participant, with color indicating chronological age. Red dashed lines indicate blood pressure values for hypertension as defined in Singapore (SBP ≥ 140 mmHg, DBP ≥ 90 mmHg), the red rectangle in the top-right corner highlights the hypertension range.
