# Supplementary material for: Vascular age estimation using a consumer wearable sleep tracker
Source: PLOS Digit Health. 2026 Mar 30;5(3):e0001329. doi: 10.1371/journal.pdig.0001329 (PMC13035161; doi:10.1371/journal.pdig.0001329)
Supplement: S8 Fig — Preprocessing and age prediction using deep learning model are illustrated. QC: Quality Check, FC: Fully Connected. (DOCX) [file pdig.0001329.s008.docx]

**S8 Fig. Summary of analysis pipeline.** Preprocessing and age prediction using deep learning model are illustrated. QC: Quality Check, FC: Fully Connected.
