# Supplementary material for: Vascular age estimation using a consumer wearable sleep tracker
Source: PLOS Digit Health. 2026 Mar 30;5(3):e0001329. doi: 10.1371/journal.pdig.0001329 (PMC13035161; doi:10.1371/journal.pdig.0001329)
Supplement: S9 Fig — Fiducial points are detected as follows: Onset: 1st sample on the waveform. Offset: Last sample on the waveform. Systolic peak: Point where 1st derivative crosses zero in positive to negative direction for the 1st time. Estimated diastolic peak: The peak of the second gaussian (which was fitted during template creation) was used as an estimated diastolic peak by adding %10 of it’s distance from the onset. Dicrotic notch: In the 3rd derivative signal, the nearest zero-crossing in the positive-to-negative direction closest to the estimated diastolic peak. Real diastolic peak: In the 1st derivative signal, the positive-to-negative zero-crossing within the segment between the dicrotic notch and the estimated diastolic peak. (DOCX) [file pdig.0001329.s009.docx]

**S9 Fig.** **Summary of fiducial point detection algorithm using pulse and its derivatives**. Fiducial points are detected as follows: Onset: 1^st^ sample on the waveform. Offset: Last sample on the waveform. Systolic peak: Point where 1^st^ derivative crosses zero in positive to negative direction for the 1^st^ time. Estimated diastolic peak: The peak of the second gaussian (which was fitted during template creation) was used as an estimated diastolic peak by adding %10 of it’s distance from the onset. Dicrotic notch: In the 3^rd^ derivative signal, the nearest zero-crossing in the positive-to-negative direction closest to the estimated diastolic peak. Real diastolic peak: In the 1st derivative signal, the positive-to-negative zero-crossing within the segment between the dicrotic notch and the estimated diastolic peak.
