# Supplementary material for: Vascular age estimation using a consumer wearable sleep tracker
Source: PLOS Digit Health. 2026 Mar 30;5(3):e0001329. doi: 10.1371/journal.pdig.0001329 (PMC13035161; doi:10.1371/journal.pdig.0001329)
Supplement: S10 Fig — After preprocessing, 165 participants had at least one 30-second window of high-quality data from both Fingertip (blue) and Ring (red), before applying data inclusion criteria. Dashed black line marks the number of pulses included into age prediction model (370 pulses). For the included 160 participants (with at least 370 pulses from each device), the median (IQR) number of total pulses per participant was 11,988 (11,092) from Fingertip and 11,727 (10,000) from Ring. b. Distribution of number of pulses included into waveform comparison across 158 participants for Fingertip (blue) and Ring (red). The median (IQR) number of pulses per participant (used in waveform comparison and PPG-features analysis) was 364 (152) for Fingertip and 367 (139) for Ring. (DOCX) [file pdig.0001329.s010.docx]

**S10 Fig.** **Distribution of total number of pulses.** **a.** After preprocessing, 165 participants had at least one 30-second window of high-quality data from both Fingertip (blue) and Ring (red), before applying data inclusion criteria. Dashed black line marks the number of pulses included into age prediction model (370 pulses). For the included 160 participants (with at least 370 pulses from each device), the median (IQR) number of total pulses per participant was 11,988 (11,092) from Fingertip and 11,727 (10,000) from Ring. **b.** Distribution of number of pulses included into waveform comparison across 158 participants for Fingertip (blue) and Ring (red). The median (IQR) number of pulses per participant (used in waveform comparison and PPG-features analysis) was 364 (152) for Fingertip and 367 (139) for Ring.
