# Supplementary material for: Vascular age estimation using a consumer wearable sleep tracker
Source: PLOS Digit Health. 2026 Mar 30;5(3):e0001329. doi: 10.1371/journal.pdig.0001329 (PMC13035161; doi:10.1371/journal.pdig.0001329)
Supplement: S1 Table — When PPG features were not-normalized for duration, CT (seconds) was significantly higher in the ring, while values for dT (seconds) were similar. (DOCX) [file pdig.0001329.s011.docx]

**S1 Table. Summary of PPG-features (un-normalized duration, in seconds)**. When PPG features were not-normalized for duration, CT (seconds) was significantly higher in the ring, while values for dT (seconds) were similar

| **PPG feature** | **Fingertip**, N = 158^1^ | **Ring**, N = 158^1^ | **p-value**^2^ |
| --- | --- | --- | --- |
| **CT (sec)** | 0.20 (0.03) | 0.23 (0.08) | <0.001 |
| **dT (sec)** | 0.31 (0.05) | 0.31 (0.05) | 0.4 |
| ^1^Median (IQR), ^2^Wilcoxon rank sum test | | | |
