# Supplementary material for: Vascular age estimation using a consumer wearable sleep tracker
Source: PLOS Digit Health. 2026 Mar 30;5(3):e0001329. doi: 10.1371/journal.pdig.0001329 (PMC13035161; doi:10.1371/journal.pdig.0001329)
Supplement: S2 Table — Bias is calculated as the difference between the ring and the fingertip sensor. LoA refers to Limits of Agreement. CT: Crest time, dT: distance between systolic and diastolic peaks, PW: Pulse width. (DOCX) [file pdig.0001329.s012.docx]

**S2 Table.** **Agreement between un-normalized PPG-features (in seconds)**. Bias is calculated as the difference between the ring and the fingertip sensor. LoA refers to Limits of Agreement. CT: Crest time, dT: distance between systolic and diastolic peaks.

| **PPG feature** | **Bias**  **(mean (SD))** | **[Lower LoA, Upper LoA]** | **Correlation** |
| --- | --- | --- | --- |
| **CT (sec)** | 0.03 (0.03) | [-0.03, 0.1] | 0.82 |
| **dT (sec)** | -0.01 (0.03) | [-0.07, 0.06] | 0.53 |
