# Supplementary material for: Vascular age estimation using a consumer wearable sleep tracker
Source: PLOS Digit Health. 2026 Mar 30;5(3):e0001329. doi: 10.1371/journal.pdig.0001329 (PMC13035161; doi:10.1371/journal.pdig.0001329)
Supplement: S3 Table — (DOCX) [file pdig.0001329.s013.docx]

**S3 Table.** **Regression models to predict CT, dT and RI for the fingertip sensor and the ring**

| **CT** | | | | | | |
| --- | --- | --- | --- | --- | --- | --- |
|  | Fingertip | | | Ring | | |
| **Characteristic** | **Beta** | **95% CI**^1^ | **p-value** | **Beta** | **95% CI**^1^ | **p-value** |
| **Age** | 0.26 | 0.21, 0.31 | <0.001 | 0.40 | 0.32, 0.48 | <0.001 |
| **Sex** |  |  | <0.001 |  |  | <0.001 |
| *Male* | — | — |  | — | — |  |
| *Female* | 5.4 | 4.0, 6.9 |  | 6.4 | 4.1, 8.8 |  |
| **BMI** | 0.05 | -0.17, 0.28 | 0.65 | -0.02 | -0.37, 0.34 | 0.93 |
| **SBP** | -0.01 | -0.08, 0.07 | 0.86 | -0.05 | -0.16, 0.07 | 0.43 |
| **DBP** | 0.16 | 0.05, 0.27 | 0.003 | 0.08 | -0.09, 0.25 | 0.34 |
| *R²* | 0.602 |  |  | 0.508 |  |  |
| *Adjusted R²* | 0.589 |  |  | 0.491 |  |  |
| *p-value* | <0.001 |  |  | <0.001 |  |  |

^1^CI = Confidence Interval

| **dT** | | | | | | |
| --- | --- | --- | --- | --- | --- | --- |
|  | Fingertip | | | Ring | | |
| **Characteristic** | **Beta** | **95% CI**^1^ | **p-value** | **Beta** | **95% CI**^1^ | **p-value** |
| **Age** | -0.24 | -0.33, -0.16 | <0.001 | -0.27 | -0.35, -0.20 | <0.001 |
| **Sex** |  |  | 0.56 |  |  | 0.069 |
| *Male* | — | — |  | — | — |  |
| *Female* | 0.75 | -1.8, 3.3 |  | -2.1 | -4.4, 0.16 |  |
| **BMI** | 0.25 | -0.13, 0.63 | 0.20 | 0.01 | -0.33, 0.35 | 0.96 |
| **SBP** | -0.12 | -0.24, 0.00 | 0.054 | -0.04 | -0.15, 0.07 | 0.45 |
| **DBP** | 0.05 | -0.13, 0.23 | 0.56 | 0.13 | -0.03, 0.30 | 0.10 |
| *R²* | 0.264 |  |  | 0.293 |  |  |
| *Adjusted R²* | 0.239 |  |  | 0.269 |  |  |
| *p-value* | <0.001 |  |  | <0.001 |  |  |
| ^1^CI = Confidence Interval | | | | | | |

| **RI** | | | | | | |
| --- | --- | --- | --- | --- | --- | --- |
|  | Fingertip | | | Ring | | |
| **Characteristic** | **Beta** | **95% CI**^1^ | **p-value** | **Beta** | **95% CI**^1^ | **p-value** |
| **Age** | 0.004 | 0.003, 0.005 | <0.001 | 0.002 | 0.000, 0.002 | 0.030 |
| **Sex** |  |  | 0.012 |  |  | 0.228 |
| *Male* | — | — |  | — | — |  |
| *Female* | 0.039 | 0.009, 0.069 |  | 0.017 | -0.011, 0.045 |  |
| **BMI** | -0.001 | -0.005, 0.004 | 0.806 | -0.002 | -0.006, 0.002 | 0.339 |
| **SBPoffice** | 0.000 | -0.002, 0.001 | 0.796 | 0.000 | -0.002, 0.001 | 0.571 |
| **DBPoffice** | 0.002 | 0.000, 0.005 | 0.028 | 0.001 | -0.001, 0.003 | 0.189 |
| *R²* | 0.372 |  |  | 0.069 |  |  |
| *Adjusted R²* | 0.351 |  |  | 0.038 |  |  |
| *p-value* | <0.001 |  |  | 0.058 |  |  |
| ^1^CI = Confidence Interval | | | | | | |
