# Supplementary material for: Vascular age estimation using a consumer wearable sleep tracker
Source: PLOS Digit Health. 2026 Mar 30;5(3):e0001329. doi: 10.1371/journal.pdig.0001329 (PMC13035161; doi:10.1371/journal.pdig.0001329)
Supplement: S4 Table — (DOCX) [file pdig.0001329.s014.docx]

**S4 Table. Age prediction performance of the PPG-based regression model using the fingertip sensor and the ring.**

|  | **Fingertip, N=158**^1^ | **Ring, N=158**^1^ | **p-value** |
| --- | --- | --- | --- |
| **MAE** | 7.76 (1.49) | 8.95 (1.54) | 0.08 |
| **RMSE** | 9.69 (2.0) | 10.8 (1.90) | 0.25 |
| **R^2^** | 0.60 (0.17) | 0.52 (0.2) | 0.66 |
| **r** | 0.77 (0.11) | 0.70 (0.14) | 0.55 |
| ^1^Mean (SD), MAE: Mean absolute error, RMSE: Root mean squared error, R^2^: Coefficient of determination, r: Correlation coefficient  Model: Age ~ CT + dT + RI | | | |
