# Supplementary figures and images for: Vascular age estimation using a consumer wearable sleep tracker
Source: PLOS Digit Health. 2026 Mar 30;5(3):e0001329. doi: 10.1371/journal.pdig.0001329 (PMC13035161; doi:10.1371/journal.pdig.0001329)

**S5 Table.** **Prediction performance for all test sets for Fingertip and Ring**


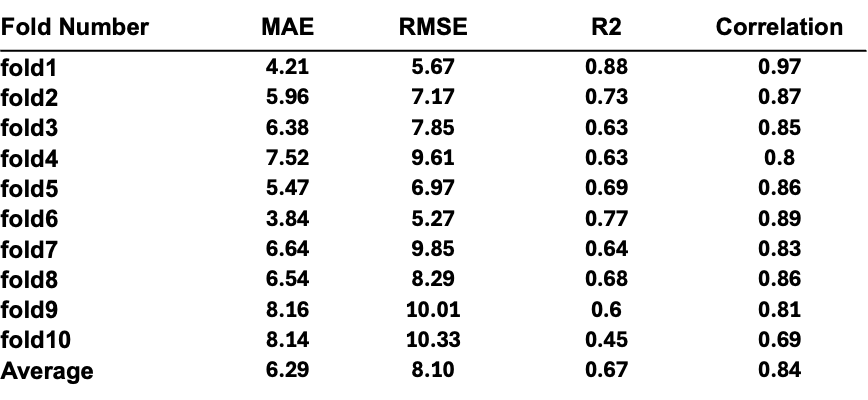


**Fingertip**


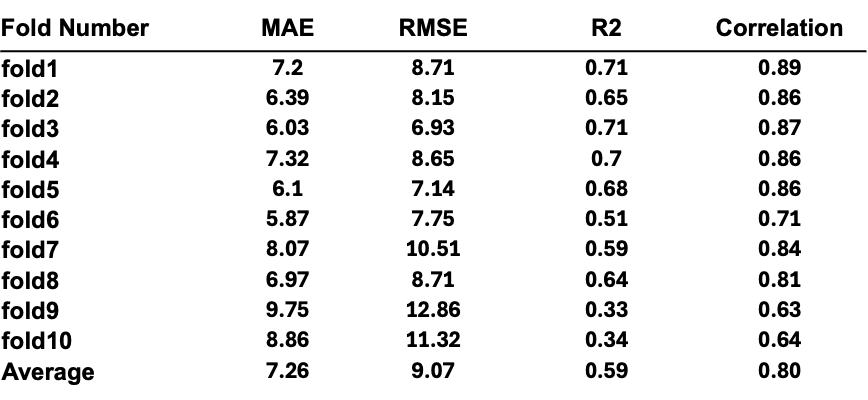


**Ring**

Supplement: S5 Table — (DOCX) [file pdig.0001329.s015.docx]
