# Supplementary material for: Vascular age estimation using a consumer wearable sleep tracker
Source: PLOS Digit Health. 2026 Mar 30;5(3):e0001329. doi: 10.1371/journal.pdig.0001329 (PMC13035161; doi:10.1371/journal.pdig.0001329)
Supplement: S6 Table — (DOCX) [file pdig.0001329.s016.docx]

**S6 Table.** **Hyperparameter values used in optimization and evaluation**.

| **Hyperparameter** | **Compared options (Test)** |
| --- | --- |
| **Dropout Rate** | 0.2, 0.4 |
| **Learning Rate** | 0.01, 0.001, 0.0001, 0.00001 |
| **Activation Function** | ReLU |
| **Optimizer** | SGD, Adam |
| **Loss Function** | L1, MSE |
| **Batch size** | 128, 256 |
| **Epoch size** | 200, 300, 500 |
